# Investigation of Dietary Intake and Nutrient Adequacy of Adolescents in Institutional Care by Comparing with National Survey Data in Taiwan: A Cross-Sectional Study

**DOI:** 10.3390/nu18111679

**Published:** 2026-05-24

**Authors:** Hsin-Nung Kao, Kuang-Shuo Chen, Tsan-Hon Liou, Ning-Jo Kao, Kai-Wei Liao, Shyh-Hsiang Lin

**Affiliations:** 1 School of Nutrition and Health Sciences, Taipei Medical University, Taipei 110, Taiwan; specialflora0905@gmail.com; 2 School of Medicine, Taipei Medical University, Taipei 110, Taiwan; 3 Department of Physical Medicine and Rehabilitation, Wan Fang Hospital, Taipei Medical University, Taipei 116, Taiwan; 4 Department of Nutrition and Health Sciences, Kainan University, Taoyuan 338, Taiwan; 5 School of Food Safety, Taipei Medical University, Taipei 110, Taiwan

**Keywords:** adolescents, residential care, nutrient adequacy, Taiwan, hidden malnutrition, health inequalities, propensity score matching

## Abstract

**Background/Objectives:** Adolescence is a critical life stage characterized by rapid growth, increased nutrient requirements, and the establishment of long-term healthy behaviors. Growing evidence suggests that nutritional inadequacies may persist even when conventional indicators such as body mass index (BMI) appear normal, reflecting hidden malnutrition, a condition characterized by micronutrient inadequacy despite adequate energy intake. This issue may be particularly relevant in structurally constrained environments. This study aimed to compare dietary intake and nutrient adequacy between adolescents residing in residential care institutions (RCIs) and those in the general population in Taiwan. **Methods:** A total of 248 adolescents were included in the analysis. Institutional data were collected in 2018 and compared with nationally representative data from the Nutrition and Health Survey in Taiwan (NAHSIT 2010–2012). To improve comparability, 1:1 propensity score matching (PSM) was applied based on age, sex, and geographic region. Nutrient intakes were evaluated according to the Taiwan Dietary Reference Intakes (DRIs). **Results:** Adolescents in RCIs demonstrated significantly lower energy and protein adequacy than their counterparts in the general population. Among boys aged 13–15 years, the proportion meeting protein adequacy was substantially lower in RCIs than in the general population (34.0% vs. 84.0%). Similarly, among girls aged 13–15 years, energy adequacy was markedly lower in RCIs (25.0% vs. 63.9%). In addition, inadequate intake of multiple micronutrients, particularly B vitamins and essential minerals, was observed. Despite these differences, BMI remained largely comparable between groups, indicating a mismatch between anthropometric status and underlying nutritional quality. **Conclusions:** These findings suggest that hidden nutritional vulnerability may persist even within structured institutional environments designed to ensure stable food provision. The results highlight the limitations of relying solely on anthropometric indicators to assess nutritional status and underscore the need for targeted nutritional strategies to improve dietary quality and reduce health inequalities in residential care settings.

## 1. Introduction

Health inequalities remain a major global public health challenge, driven by complex interactions among social, economic, and environmental determinants [1,2,3]. These disparities are increasingly understood as being embedded within structural contexts—such as food environments and access to resources—rather than arising solely from individual behaviors [1,2,3]. Nutrition represents a key pathway through which such inequalities become biologically embedded across the life course [1,2,3].

Adolescence is a critical developmental stage characterized by rapid growth, increased nutrient requirements, and the establishment of long-term healthy behaviors [4,5,6,7]. Adequate intake of key nutrients—including protein, calcium, iron, and zinc—is essential to support growth, bone mineralization, hematopoiesis, and immune function [5,6,7]. Inadequate intake during this period may impair development and increase vulnerability to adverse health outcomes [4,5,6,7].

Despite these physiological demands, adolescents are increasingly exposed to poor diet quality, micronutrient deficiencies, and rising rates of overweight and obesity [8,9,10]. The double burden of malnutrition (DBM) is increasingly understood as being driven by structural and environmental factors, including adverse food environments and limited dietary diversity, rather than solely individual behaviors [9]. Such conditions may promote the consumption of energy-dense but nutrient-poor foods, contributing to the coexistence of excess energy intake and micronutrient deficiencies. Importantly, conventional indicators such as body mass index (BMI) may not adequately capture underlying nutritional status [11,12,13]. Adolescents may present with normal or elevated BMI while experiencing substantial micronutrient deficiencies, a condition referred to as “hidden malnutrition” [11,12,13,14]. This mismatch between energy intake and nutrient quality may adversely affect cognitive, immune, and metabolic health, with potential long-term consequences [13,14].

Globally, adolescents from socioeconomically disadvantaged backgrounds consistently exhibit poorer dietary quality, with lower intake of nutrient-dense foods and higher consumption of energy-dense, nutrient-poor foods [14,15,16,17]. These patterns are shaped by structural constraints, including food insecurity and limited dietary diversity [14,15,16].

Residential care institutions (RCIs) represent a unique and understudied setting in which dietary intake is largely determined by centralized food systems [18,19,20,21]. Previous studies have reported that adolescents in such settings often experience imbalanced dietary patterns, including insufficient intake of protein and key micronutrients such as iron, calcium, and B vitamins [18,19,20,21].

However, these studies are limited by heterogeneous study designs, relatively small sample sizes, and a focus on selected nutrients, which may restrict the generalizability of their findings [18,19,20,21]. In addition, inconsistencies in dietary assessment methods and population characteristics make direct comparisons across studies difficult [18,19,20,21]. Despite these limitations, the available evidence consistently suggests suboptimal nutrient adequacy among institutionalized adolescents.

Furthermore, most existing studies have focused on limited nutrients or specific subgroups, and comprehensive comparisons with nationally representative populations remain scarce [18,19,20,21]. These structural and environmental constraints may be particularly relevant in residential care settings, where food provision is structured but may not fully meet adolescents’ nutritional needs.

In Taiwan, national surveys such as the Nutrition and Health Survey in Taiwan (NAHSIT) provide representative data for the general population [22,23,24]. However, comparable evidence for adolescents in RCIs is scarce.

Therefore, this study aimed to compare dietary intake and nutrient adequacy between adolescents in RCIs and those in the general population in Taiwan. This study provides novel evidence by examining hidden nutritional vulnerability within institutional care settings, where food environments are structured yet may not ensure adequate nutritional quality.

## 2. Materials and Methods

### 2.1. Study Population

Adolescents aged 10–18 years were recruited from residential care institutions (RCIs) in Taiwan. A list of eligible institutions was obtained from the Social and Family Affairs Administration. Four institutions were randomly selected across Northern, Central, and Eastern regions to improve geographic representation. All eligible adolescents were invited to participate, and recruitment within institutions followed a convenience sampling approach based on participants’ availability and consent. Participation was voluntary, and written informed consent was obtained from legal guardians.

### 2.2. Control Group and Propensity Score Matching

To establish a comparable control group, adolescents within the same age range were selected from the NAHSIT 2010–2012 dataset. PSM was applied to reduce confounding and improve comparability between groups. Detailed procedures are described in Section 2.6.

### 2.3. Study Design and Data Sources

This cross-sectional comparative study evaluated dietary intake among adolescents in RCIs and compared it with that of the general population. Data were obtained from primary data collected in RCIs between 10 January 2018 and 30 June 2018 and secondary data from the Nutrition and Health Survey in Taiwan (NAHSIT) 2010–2012. NAHSIT is a nationwide survey using a multistage stratified cluster sampling design to provide nationally representative data [22]. Demographic characteristics, dietary intake, anthropometric measurements, and health-related indicators were collected. Adolescents within the target age range were selected from NAHSIT as the comparison group.

To improve comparability between groups and reduce potential selection bias, propensity score matching (PSM), a statistical method that balances baseline characteristics across observational groups, was performed using age, sex, and geographic region as covariates. Matching was conducted using nearest-neighbor matching without replacement, with a caliper width equal to 0.2 times the propensity score’s logit standard deviation. The geographic region was harmonized across datasets by restricting the analysis to comparable strata (North, Central, and East), corresponding to strata B, C, and E in NAHSIT. Covariate balance was assessed using standardized mean differences (SMDs), with values < 0.1 indicating acceptable balance. Detailed covariate balance results before and after matching are presented in Supplementary Table S1. After matching, 248 matched pairs (496 participants) were retained for analysis.

### 2.4. Dietary and Nutrient Intake Calculation

Dietary intake in RCIs was assessed using an interviewer-administered 24 h dietary recall approach adapted to institutional meal settings. Institutional menus were obtained to characterize the foods provided. Meals were generally standardized with limited individual choice, and individual intake was assessed through participant interviews regarding the actual amount of food consumed at each meal. Dietary intake was assessed based on meals consumed on a single survey day. Daily nutrient intake was calculated using Nutritionist Edition software, Version III (Ekitchen Business Corp., Taichung, Taiwan). The software incorporates food composition data reflecting Taiwanese dietary patterns and information relevant to the study period and is commonly used in nutrition research and practice in Taiwan. The analysis included energy intake (kcal), macronutrients (protein, fat, and carbohydrates), and selected vitamins and minerals. Nutrient adequacy was evaluated according to sex- and age-specific Taiwan Dietary Reference Intakes (DRIs, 8th edition, 2022) [25]. For the comparison group, dietary intake data were obtained from the Nutrition and Health Survey in Taiwan (NAHSIT) 2010–2012, which employed standardized 24 h dietary recall interviews conducted by trained interviewers in a free-living population. Although NAHSIT also included food frequency questionnaire (FFQ) components, only the standardized 24 h dietary recall data were used for the present comparison.

### 2.5. Nutrient Adequacy Definition and Heatmap Analysis

Participants were stratified by sex and age group (10–12, 13–15, and 16–18 years) to assess nutrient intake patterns. For each stratum, nutrient adequacy and sodium intake distribution were separately evaluated between the RCI and NAHSIT groups. For most nutrients, adequacy was defined as intake meeting or exceeding the recommended levels from the Taiwan DRIs.

For sodium, intake was evaluated relative to the Tolerable Upper Intake Level (UL). The proportion of participants with intake ≤ UL was calculated to indicate the prevalence of non-excessive sodium intake. As the UL is designed to reflect the risk of excessive intake, it does not represent nutritional adequacy. Accordingly, a higher percentage reflects a greater proportion of individuals with non-excessive sodium intake.

Nutrient adequacy was visualized using heatmaps. Panel A shows adequacy percentages for the residential care institutions. Panel B shows adequacy percentages for the NAHSIT group. Panel C presents the absolute difference in percentage points (pp), calculated as:

Difference (pp) = RCIs (%) − NAHSIT (%)

Positive values indicate higher adequacy in the RCIs. Negative values indicate lower adequacy compared with the NAHSIT group. Differences across nutrients and strata were assessed. Multiple comparisons were adjusted using the Benjamini–Hochberg false discovery rate (BH-FDR) procedure, with statistical significance defined as q < 0.05. Heatmaps were generated in R (version 4.1.1) using the ggplot2 (version 4.0.3) and pheatmap (version 1.0.13) packages.

### 2.6. Propensity Score Matching

To minimize baseline differences between adolescents in RCIs and those in the general population, PSM was performed. Propensity scores were estimated using a logistic regression model, with group membership (RCIs vs. the general population) as the dependent variable and age, sex, and geographic region (Northern, Central, and Eastern Taiwan) as covariates. These variables were selected based on their availability in both datasets and their known associations with dietary intake and nutritional status. A 1:1 nearest-neighbor matching without replacement was applied, with a caliper width equal to 0.2 times the standard deviation of the logit of the propensity score. Only successfully matched pairs were retained for subsequent analyses. Balance between groups after matching was assessed using standardized mean differences (SMD), with values < 0.1 indicating adequate balance. All PSM procedures were conducted using R software (version 4.1.1). A total of 248 matched pairs were retained after matching.

Although propensity score matching improves comparability between groups, residual confounding from unmeasured variables (e.g., socioeconomic status, family environment, and lifestyle factors) cannot be ruled out [26,27]. In addition, the matched sample may not fully retain the national representativeness of the original survey dataset.

### 2.7. Statistical Analysis

Statistical analyses were conducted using IBM SPSS Statistics (version 25; IBM Corp., Armonk, NY, USA), with propensity score matching performed in R (version 4.1.1). Continuous variables are presented as mean ± standard deviation, and categorical variables as frequencies and percentages. Group differences were assessed using independent-samples *t* tests (with Welch’s correction when variances were unequal); when ANOVA indicated significant differences, post hoc tests were applied based on the assumption of equal or unequal variances, as determined by Levene’s test. Categorical variables were analyzed using the chi-square test.

For comparisons involving small expected cell counts, Fisher’s exact test was applied.

To address multiple comparisons, the Benjamini–Hochberg false discovery rate (BH-FDR) procedure was applied to the nutrient adequacy analyses (global adjustment). No adjustment for multiple comparisons was applied to Table 1 and Table 2, as these analyses were primarily descriptive. All statistical tests were two-tailed. A *p*-value < 0.05 was considered statistically significant for analyses without multiple comparison adjustment (Table 1 and Table 2), whereas for nutrient adequacy analyses (Table 3), statistical significance was determined after BH-FDR correction (q < 0.05).

### 2.8. Ethical Approval

The study was approved by the Taipei Medical University Joint Institutional Review Board (TMU-JIRB) (Approval No. N201712041). Data collection was conducted between January and June 2018. All data were analyzed in de-identified forms to ensure participant confidentiality.

## 3. Results

### 3.1. Baseline Characteristics of Adolescents in Residential Care Institutions

As shown in Table 1, baseline characteristics were generally comparable across regions. A total of 248 adolescents residing in RCIs were included in the analysis, with 101 (40.7%), 82 (33.1%), and 65 (26.2%) from Northern, Central, and Eastern Taiwan, respectively. The overall sample comprised 127 boys (51.2%) and 121 girls (48.8%). The distribution of sex varied significantly across regions (χ^2^ = 7.06, *p* = 0.029). The overall mean age was 14.20 ± 2.20 years, with no significant regional difference (*p* = 0.990). Anthropometric indicators were comparable across regions. Mean height (*p* = 0.685), weight (*p* = 0.149), and body mass index (BMI) (*p* = 0.061) did not differ significantly among the three regions. However, adolescents in Eastern Taiwan had a numerically higher mean BMI (20.83 ± 3.92 kg/m^2^) than those in Northern and Central Taiwan. Regarding BMI categories, 11.7% of participants were underweight, 66.5% were normal weight, 11.3% were overweight, and 10.5% were obese. The distribution of BMI categories did not differ significantly across regions (χ^2^ = 0.245). All expected cell counts met the assumptions for the chi-square test (minimum expected count = 6.81). The linear-by-linear association test for ordinal BMI categories was not statistically significant (*p* = 0.063).

### 3.2. Baseline Characteristics After Propensity Score Matching

After propensity score matching, 248 matched pairs were retained for analysis. Baseline characteristics between adolescents in RCIs and the NAHSIT group were well balanced (Table 1). Covariate balance was improved after matching, with all standardized mean differences below 0.1 (Supplementary Table S1).

### 3.3. Sex-Stratified Differences in Dietary Intake

As shown in Table 2, boys residing in RCIs had significantly lower total energy intake than boys in the NAHSIT dataset (*p* < 0.001). Intakes of protein, fat, carbohydrates, and all major fatty acid subtypes were significantly lower in RCIs (all *p* < 0.05). Most B vitamins, including thiamin, riboflavin, niacin, vitamin B6, and vitamin B12, as well as several minerals, were significantly lower in RCIs (all *p* < 0.001). In contrast, vitamin A, vitamin D, and vitamin E intakes did not differ significantly between groups. Sodium and cholesterol intakes were markedly lower in RCIs (both *p* < 0.001).

Girls residing in RCIs also had significantly lower total energy intake than those in the NAHSIT dataset (*p* = 0.008). Protein intake was significantly lower (*p* < 0.001), whereas total fat, fatty acid subtypes, and carbohydrate intake did not significantly differ between groups. Dietary fiber intake was significantly higher in RCIs (*p* = 0.003). For micronutrients, intakes of vitamin A, vitamin D, vitamin E, vitamin B1, niacin, vitamin B6, and vitamin B12 were significantly lower in girls residing in RCIs (all *p* < 0.05). In contrast, vitamin B2, vitamin C, iron, magnesium, and calcium did not differ significantly between groups. Potassium, phosphorus, zinc, sodium, and cholesterol intakes were significantly lower in RCIs (all *p* < 0.05) (Table 2).

### 3.4. DRI Adequacy of Energy and Macronutrients

**Figure 1:**
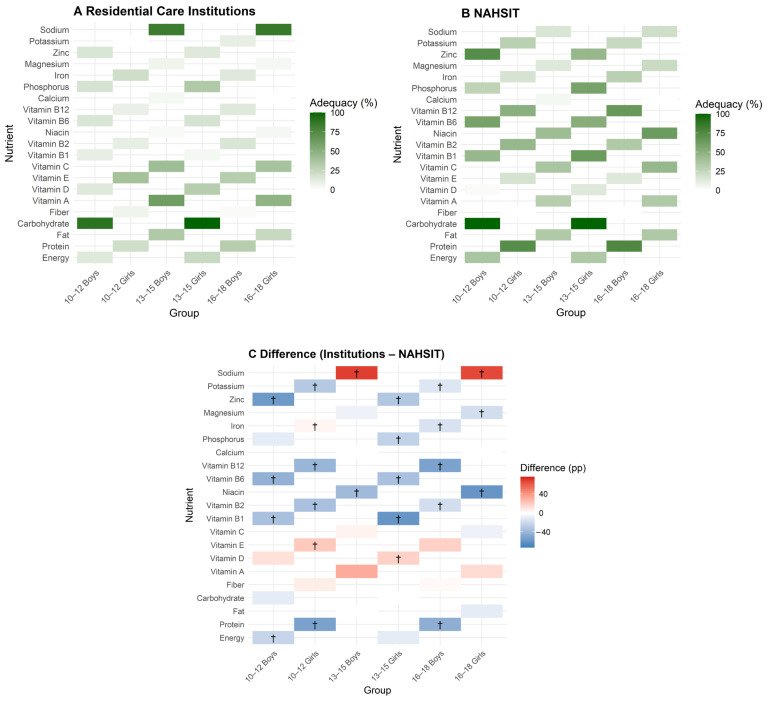
Heatmaps of nutrient adequacy among adolescents in RCIs and the NAHSIT comparison group stratified by sex and age group. (**A**) Nutrient adequacy in RCIs. (**B**) Nutrient adequacy in the NAHSIT group. (**C**) Differences in adequacy percentages (RCIs − NAHSIT). Negative values indicate lower adequacy in the RCI group compared with the NAHSIT group. Separate color scale legends are displayed alongside each panel for clarity. † Indicates statistically significant differences after BH–FDR correction (q < 0.05).

Figure 1 illustrates nutrient adequacy across sex and age groups for adolescents in RCIs (Figure 1A), the general population (NAHSIT; Figure 1B), and the corresponding between-group differences (Figure 1C). For macronutrients, carbohydrate adequacy was consistently high across all sex–age strata in both groups. In contrast, dietary fiber adequacy remained uniformly low in both populations, as indicated by lower adequacy levels in Figure 1A,B. Energy and protein adequacy were moderate overall, with generally higher levels in the NAHSIT group than in the RCIs, consistent with the negative differences shown in Figure 1C. Fat intake (within the AMDR range), carbohydrate adequacy, and dietary fiber adequacy exhibited relatively small between-group differences across most strata. Overall, patterns of macronutrient adequacy were broadly similar across sex and age groups, with limited variability between RCIs and the general population. In contrast, more pronounced differences in micronutrient adequacy were observed between the two groups.

### 3.5. DRI Adequacy of Micronutrients

For micronutrients, several nutrients showed consistently lower adequacy in RCIs across nearly all sex–age strata. These included vitamin B1, vitamin B2, niacin, vitamin B6, vitamin B12, phosphorus, magnesium, zinc, and potassium, all of which showed lower adequacy levels in Figure 1A than in Figure 1B, with corresponding negative differences in Figure 1C. Among these, niacin and vitamin B1 exhibited the largest deficits, reaching differences of up to approximately −70 percentage points in certain strata, as detailed in Table 3. Calcium and iron also demonstrated generally lower adequacy in RCIs, although the magnitude of differences varied across sex and age groups. Vitamin A, vitamin C, and vitamin E showed moderate adequacy in both groups, whereas vitamin D adequacy remained low across most strata. Although some positive differences were observed in Figure 1C for specific subgroups, this pattern was not consistent across all sex–age strata. In contrast, sodium adequacy (defined as intake ≤ UL) was high in both groups (Figure 1A,B) and showed consistently large positive differences in Figure 1C, with differences ranging from approximately +61 to +76 percentage points in Table 3, indicating a higher proportion of adolescents meeting sodium adequacy criteria in RCIs. Across sex and age categories, similar patterns were observed, with no subgroup demonstrating a consistently distinct trend, suggesting that the observed differences were broadly uniform across strata. Detailed numerical values and statistical comparisons corresponding to these patterns are presented in Table 3.

## 4. Discussion

### 4.1. Principal Findings Within a Structural Framework

Adolescents residing in RCIs exhibited lower energy and protein intake and reduced adequacy of multiple micronutrients across sex–age strata. Notably, these deficits were not reflected in anthropometric indicators, highlighting the limited sensitivity of BMI in detecting underlying nutritional inadequacies. This pattern is consistent with the concept of hidden malnutrition, where individuals may appear nutritionally normal despite significant micronutrient deficiencies [11,12,13,28]. The consistency of these inadequacies across strata suggests that the observed patterns are unlikely to be driven solely by individual variation but rather reflect structural characteristics of institutional food provision. These findings align with the social determinants of health framework, indicating that nutritional outcomes are shaped by environmental and systemic constraints rather than by individual dietary choices alone [1,2,3,18,19,20,21].

### 4.2. Micronutrient Intake and Nutritional Quality

The observed inadequacy of B vitamins, iron, and zinc is consistent with previous evidence showing that adolescents frequently fail to meet micronutrient requirements, particularly in settings with limited dietary diversity [11,12,14,28]. These nutrients play important roles in energy metabolism, hematopoiesis, immune function, and growth, and deficiencies during adolescence may have both immediate and long-term health consequences [4,5,6,7]. Rather than representing isolated deficiencies, these findings likely reflect broader dietary patterns characterized by adequate energy intake but poor dietary quality. Diets high in refined carbohydrates and low in dairy and animal-source foods may contribute to these deficiencies and reinforce underlying nutritional vulnerability.

Such patterns are consistent with the nutrition transition, in which energy-dense, nutrient-poor diets contribute to declining dietary quality [8,9,10]. Greater reliance on refined and ultra-processed foods may further contribute to deficiencies in B vitamins, including thiamin and niacin [23,29]. Comparable dietary imbalances and suboptimal nutritional patterns have also been reported among adolescents in other Asian countries, including China [30], suggesting that these nutritional patterns may reflect broader structural characteristics of dietary systems in institutional or resource-constrained settings across the region.

Suggesting that these nutritional patterns may reflect broader structural characteristics of dietary systems in institutional or resource-constrained settings across the region.

The markedly lower sodium intake observed in the RCIs group compared with the NAHSIT group should be interpreted cautiously. Although differences in institutional meal provision and food environments may partly contribute to lower sodium intake, methodological differences between datasets may also have influenced sodium estimation, particularly for condiments, sauces, and processed foods. Moreover, sodium intake below the UL should not necessarily be interpreted as optimal, as excessively low intake may affect electrolyte and fluid balance.

### 4.3. Mechanisms Linking Institutional Environments to Nutritional Inequality

The observed disparities may be influenced by multiple factors related to institutional environments. Structurally, institutional food systems often prioritize cost efficiency and standardization, potentially limiting dietary diversity and access to nutrient-dense foods [19,20,21]. Institutional meal structures may rely more heavily on refined staple foods while providing limited amounts of protein-rich and animal-source foods, such as meat, eggs, legumes, and dairy products, which are important sources of B vitamins, iron, zinc, and calcium. Lower dietary diversity and reduced whole-grain intake may further contribute to the observed nutritional inadequacies.

Environmentally, fixed meal schedules and restricted food choices may constrain dietary intake patterns. Psychosocially, reduced autonomy and environmental stress may further influence eating behaviors. Together, these factors suggest that systemic constraints within institutional environments may contribute to nutritional inequalities in this population, although individual-level factors may also play a role.

### 4.4. Interpretation of Nutrient Patterns and Food Sources

Although this study focused on nutrient-level analysis, the observed patterns may provide indirect insights into underlying food group consumption. For example, lower intakes of calcium and vitamin D may indicate insufficient dairy intake, whereas lower vitamin C intake and generally suboptimal dietary fiber intake may reflect limited fruit and vegetable consumption. Similarly, lower intakes of iron, zinc, and B vitamins may suggest reduced consumption of animal-source foods.

To facilitate interpretation of the nutrient-level findings presented in the heatmap, Supplementary Table S2 provides a conceptual mapping between selected nutrient patterns and their potential food sources. While the heatmap summarizes differences in nutrient adequacy, the Supplementary Table contextualizes these findings within possible dietary patterns. However, because detailed dietary pattern and food source analyses were not performed, these interpretations should be considered exploratory and interpreted with caution.

Future studies incorporating food-based dietary assessment and dietary pattern analysis are warranted to better inform targeted nutritional interventions.

### 4.5. Policy and Nutritional Implications

Although institutional meal systems ensure food provision, they may not adequately address dietary quality and micronutrient adequacy. Developing standardized menu guidelines that incorporate protein-rich and micronutrient-dense foods may improve dietary quality in institutional settings [31,32]. In addition, strategies such as micronutrient fortification and targeted supplementation programs may be effective in addressing nutritional deficiencies, particularly in resource-constrained settings [11,12,28]. Routine nutritional screening and dietary monitoring programs may help identify at-risk adolescents and guide targeted interventions.

### 4.6. Life-Course Implications

Adolescence is a critical period for establishing long-term health trajectories [4,5,6,7,13]. The observed inadequacies in energy, protein, and micronutrient intake may have cumulative effects across the life course, increasing the risk of impaired development and chronic disease in adulthood [13,33]. Importantly, these deficits may not be apparent from anthropometric measures alone. These findings suggest that nutritional inequalities during adolescence may contribute to persistent health disparities and, over time, reinforce broader social inequities [1,2,3,13].

### 4.7. Strengths and Limitations

This study provides a comprehensive evaluation of dietary intake and nutrient adequacy among adolescents in RCIs, a relatively understudied population. The use of a nationally representative comparison group and propensity score matching strengthens the validity of the findings. Several limitations should be acknowledged. First, the institutional dataset was collected approximately 6–8 years after the NAHSIT 2010–2012 survey, which may limit temporal comparability between groups. Previous Taiwanese studies have reported increasing consumption of ultra-processed and energy-dense foods among adolescents over time, accompanied by changes in dietary quality and dietary patterns [23]. Therefore, some observed differences may partially reflect secular dietary trends rather than institutional effects alone. Second, the propensity score-matching model included only age, sex, and geographic region because these variables were consistently available across both datasets. Important variables, such as socioeconomic status, physical activity, urban–rural residence, and prior dietary habits, were not consistently available and therefore could not be included in the matching process. Although propensity score matching improved comparability between groups, residual confounding from unmeasured variables may persist and influence the observed differences. Accordingly, causal inferences should be avoided. In addition, participation in this study was voluntary, and some institutions declined participation during recruitment, which may further limit the representativeness of the sample. Third, dietary intake was assessed using different data-collection approaches across datasets, which may introduce measurement variability and potential measurement bias. In addition, reliance on self-reported dietary data may lead to recall bias, and the absence of biochemical validation (e.g., serum ferritin or 25-hydroxyvitamin D) may limit the ability to fully assess micronutrient status. This limitation is particularly relevant for vitamin D, for which circulating levels are strongly influenced by sun exposure and may not be adequately reflected by dietary intake alone. In addition, participation was voluntary, which may have introduced selection bias if adolescents who agreed to participate differed systematically from those who did not. Furthermore, dietary intake in RCIs was based on a single survey day and may therefore not fully reflect usual intake due to day-to-day and seasonal variation, particularly for micronutrients with high within-person variability. In addition, folate intake was not analyzed because of limitations in the available dietary dataset and inconsistencies in food composition data, which may affect measurement reliability. Finally, dietary intake was analyzed using a food composition database adapted for Taiwanese dietary patterns; however, potential discrepancies between the database and the time of data collection should be considered when interpreting the results. In addition, some sex- and age-stratified subgroup analyses involved relatively small sample sizes, which may have limited statistical power. In addition, sensitivity analyses using alternative matching approaches or exclusion of extreme intake values were not performed in the present study. We are currently planning future studies incorporating additional analytical approaches to further evaluate the robustness of the observed findings. Despite these limitations, the findings highlight the coexistence of inadequate nutrient intake and relatively normal anthropometric status, consistent with the broader framework of the double burden of malnutrition [8,9,10].

## 5. Conclusions

In conclusion, adolescents residing in residential care institutions (RCIs) demonstrated lower adequacy of several key nutrients, particularly energy, protein, vitamin B complex, zinc, potassium, and calcium, despite generally normal anthropometric status. These findings suggest the presence of hidden nutritional vulnerability that may not be identifiable through conventional growth indicators alone. Addressing these nutritional gaps may require integrated, multi-level strategies to improve dietary quality, enhance nutrient density, and strengthen routine nutritional monitoring within institutional settings. Targeted nutritional interventions may help reduce nutritional inequalities and support long-term health outcomes in this vulnerable population. Future research should incorporate longitudinal study designs to better understand the long-term implications of these nutritional patterns. In addition, where feasible and ethically appropriate, studies incorporating biochemical assessments may help validate dietary intake and provide a more comprehensive evaluation of nutrient status.

## Figures and Tables

**Table 1:** Baseline characteristics of adolescents in residential care institutions in Taiwan.

	North	Central	East	Total	*p*-Value
*N*	101 (40.7)	82 (33.1)	65 (26.2)	248 (100.0)	
Sex, *n* (%)					0.029
Boys	52 (40.9)	51 (40.2)	24 (18.9)	127 (51.2)	
Girls	49 (40.5)	31 (25.6)	41 (33.9)	121 (48.8)	
Age (years)	14.22 ± 2.37	14.21 ± 2.08	14.17 ± 2.12	14.20 ± 2.20	0.990
Height (cm)	154.57 ± 11.85	155.72 ± 10.81	155.95 ± 10.73	155.31 ± 11.20	0.685
Weight (kg)	47.43 ± 12.51	48.79 ± 11.84	51.31 ± 13.15	48.90 ± 12.51	0.149
BMI (kg/m^2^)	19.55 ± 3.21	19.89 ± 3.24	20.83 ± 3.92	20.00 ± 3.45	0.061
BMI category					0.245
Underweight, *n* (%)	15 (14.9)	8 (9.8)	6 (9.2)	29 (11.7)	
Normal, *n* (%)	67 (66.3)	60 (73.2)	38 (58.5)	165 (66.5)	
Overweight, *n* (%)	10 (9.9)	6 (7.3)	12 (18.5)	28 (11.3)	
Obesity, *n* (%)	9 (8.9)	8 (9.8)	9 (13.8)	26 (10.5)	

Values are presented as *n* (%). Percentages were calculated within each row. Between-group differences across regions were assessed using Pearson’s chi-square test. *p*-value < 0.05 was considered statistically significant.

**Table 2:** Dietary intake of energy and nutrients in residential care institutions and the NAHSIT population in Taiwan.

	Boys				Girls			
Nutrient	(RCIs, *n* = 127)	(NAHSIT, *n* = 127)	Mean Difference (95% CI)	*p*-Value	(RCIs, *n* = 121)	(NAHSIT, *n* = 121)	Mean Difference (95% CI)	*p*-Value
**Energy (kcal/day)**	1963 ± 676	2699 ± 1066	−736 (−957, −514)	<0.001	1775 ± 656	2035 ± 848	−260 (−453, −68)	0.008
**Protein (g/day)**	56.1 ± 21.5	106.3 ± 46.4	−50.2 (−59.1, −41.2)	<0.001	50.9 ± 18.1	76.7 ± 34.0	−25.9 (−32.8, −19.0)	<0.001
Fat (g/day)	79.1 ± 34.4	102.50 ± 58.5	−23.4 (−35.2, −11.5)	<0.001	70.1 ± 27.1	78.1 ± 43.5	−8.6 (−17.8, 0.6)	0.066
Monounsaturated fatty acids (g/day)	26.5 ± 15.7	36.0 ± 23.0	−9.4 (−14.3, −4.6)	<0.001	24.9 ± 13.6	28.1 ± 17.3	−3.2 (−7.2, 0.7)	0.109
Polyunsaturated fatty acids (g/day)	24.8 ± 17.3	31.0 ± 19.6	−6.2 (−10.8, −1.7)	0.008	23.6 ± 14.2	22.3 ± 13.5	1.3 (−2.2, 4.8)	0.456
Saturated fatty acids (g/day)	14.1 ± 13.5	35.4 ± 22.5	−21.2 (−25.8, −16.6)	<0.001	13.5 ± 19.1	26.7 ± 16.9	−13.2 (−17.8, −8.7)	<0.001
Carbohydrates (g/day)	256.7 ± 94.8	340.0 ± 142.9	−83.3 (−113.3, −53.4)	<0.001	235.1 ± 107.3	257.8 ± 109.2	−22.7 (−50.1, 4.7)	0.104
**Dietary fiber (g/day)**	16.5 ± 8.3	13.4 ± 8.7	3.1 (1.0, 5.2)	0.004	15.6 ± 8.2	12.3 ± 8.8	3.2 (1.1, 5.4)	0.003
Vitamin A (µg RE/day)	621.8 ± 481.2	686.6 ± 479.9	−64.8 (−183.6, 54.0)	0.283	644.0 ± 606.8	845.7 ± 441.6	−201.7 (−336.2, −67.2)	0.003
Vitamin D (µg/day)	5.0 ± 10.7	6.4 ± 7.7	−1.4 (−3.7, 0.9)	0.240	4.7 ± 5.6	8.1 ± 10.7	−3.3 (−5.5, −1.1)	0.003
Vitamin E (mg α-TE/day)	9.0 ± 4.1	10.1 ± 6.9	−1.1 (−2.5, 0.2)	0.117	7.8 ± 5.8	14.0 ± 4.7	−6.3 (−7.6, −4.9)	<0.001
Vitamin C (mg/day)	83.8 ± 71.5	125.9 ± 126.7	−42.1 (−67.6, −16.6)	0.001	114.1 ± 73.7	126.7 ± 120.3	−12.6 (−37.9, 12.7)	0.328
**Vitamin B1 (mg/day)**	1.04 ± 0.56	1.87 ± 1.17	−0.83 (−1.05, −0.60)	<0.001	1.04 ± 0.54	1.24 ± 0.77	−0.20 (−0.37, −0.03)	0.020
Vitamin B2 (mg/day)	0.97 ± 0.59	1.53 ± 0.74	−0.56 (−0.72, −0.39)	<0.001	1.39 ± 0.59	1.35 ± 0.73	−0.04 (−0.13, 0.21)	0.650
**Niacin (mg/day)**	8.9 ± 3.7	23.7 ± 11.7	−14.8 (−16.9, −12.6)	<0.001	8.4 ± 3.4	18.3 ± 11.2	−9.3 (−11.4, −7.1)	<0.001
Vitamin B6 (mg/day)	1.03 ± 0.48	2.02 ± 1.03	−0.99 (−1.19, −0.79)	<0.001	1.35 ± 0.48	1.63 ± 1.00	−0.28 (−0.48, −0.08)	0.006
Vitamin B12 (µg/day)	1.5 ± 1.0	6.1 ± 8.9	−4.7 (−6.3, −3.1)	<0.001	1.7 ± 1.3	4.4 ± 5.4	−2.9 (−3.9, −1.9)	<0.001
**Calcium (mg/day)**	364 ± 194	562 ± 354	−197 (−268, −127)	<0.001	442 ± 237	451 ± 282	−11 (−46, 18)	0.784
Iron (mg/day)	8.7 ± 6.7	19.7 ± 9.7	−11.1 (−13.1, −9.0)	<0.001	11.2 ± 7.6	14.6 ± 7.1	−3.4 (−5.5, −1.4)	<0.001
**Potassium (mg/day)**	1710 ± 700	2724 ± 1200	−1013 (−1257, −770)	<0.001	1717 ± 1041	2195 ± 1139	−376 (−652, −99)	<0.001
Magnesium (mg/day)	154 ± 66	297 ± 136	−143 (−169, −116)	<0.001	193 ± 79	231 ± 117	−16 (−41, 9)	0.198
Phosphorus (mg/day)	556 ± 191	1491 ± 676	−935 (−1058, −812)	<0.001	669 ± 255	1090 ± 495	−259 (−357, −160)	<0.001
Zinc (mg/day)	6.7 ± 2.7	14.2 ± 6.5	−7.4 (−8.7, −6.2)	<0.001	7.2 ± 3.4	9.9 ± 4.8	−2.6 (−3.7, −1.6)	<0.001
**Sodium (mg/day)**	1616 ± 1008	5252 ± 2878	−3636 (−4159, −3113)	<0.001	1428 ± 841	3894 ± 2312	−2107 (−2561, −1654)	<0.001
Cholesterol (mg/day)	209 ± 166	479 ± 324	−270 (−334, −206)	<0.001	231 ± 187	387 ± 256	−113 (−169, −58)	<0.001

Values are presented as mean ± SD. Vitamin A intake was expressed as retinol equivalents (RE) according to the food composition database used for nutrient analysis. Between-group comparisons (RCIs vs. NAHSIT) were performed using independent-samples *t*-tests (two-sided), with Welch’s correction applied when variances were unequal. Mean differences were calculated as Shelters minus NAHSIT values. RCIs, residential care institutions; NAHSIT, Nutrition and Health Survey in Taiwan.

**Table 3:** Summary of nutrient adequacy (%) in residential care institutions and NAHSIT groups for key clinically relevant nutrients.

Nutrient	Criterion	Boys (10–12 Years)			Girls (10–12 Years)			Boys (13–15 Years)			Girls (13–15 Years)			Boys (16–18 Years)			Girls (16–18 Years)		
		RCI	NAHSIT	*p*	RCI	NAHSIT	*p*	RCI	NAHSIT	*p*	RCI	NAHSIT	*p*	RCI	NAHSIT	*p*	RCI	NAHSIT	*p*
Energy	≥EER	7/33 (21.2%)	13/33 (39.4%)	0.180	6/41 (14.6%)	9/41 (22.0%)	0.569	6/50 (12.0%)	28/50 (56.0%)	<0.001	12/51 (23.5%)	16/51 (31.4%)	0.506	6/44 (13.6%)	16/44 (36.4%)	0.025	7/29 (24.1%)	10/29 (34.5%)	0.565
Protein	≥RDA	14/33 (42.4%)	27/33 (81.8%)	0.002	19/41 (46.3%)	31/41 (75.6%)	0.012	17/50 (34.0%)	42/50 (84.0%)	<0.001	18/51 (35.3%)	34/51 (66.7%)	0.003	9/44 (20.5%)	32/44 (72.7%)	<0.001	9/29 (31.0%)	22/29 (75.9%)	0.001
Fat	AMDR 25–35%	10/33 (30.3%)	13/33 (39.4%)	0.606	12/41 (29.3%)	18/41 (43.9%)	0.252	19/50 (38.0%)	21/50 (42.0%)	0.838	18/51 (35.3%)	17/51 (33.3%)	1.000	15/44 (34.1%)	15/44 (34.1%)	1.000	7/29 (24.1%)	10/29 (34.5%)	0.565
Carbohydrate	≥EAR	33/33 (100.0%)	32/33 (97.0%)	1.000	41/41 (100.0%)	41/41 (100.0%)	1.000	50/50 (100.0%)	50/50 (100.0%)	1.000	51/51 (100.0%)	49/51 (96.1%)	0.495	44/44 (100.0%)	44/44 (100.0%)	1.000	26/29 (89.7%)	29/29 (100.0%)	0.237
Dietary fiber	≥AI	2/33 (6.1%)	1/33 (3.0%)	1.000	1/41 (2.4%)	1/41 (2.4%)	1.000	1/50 (2.0%)	0/50 (0.0%)	1.000	3/51 (5.9%)	2/51 (3.9%)	1.000	1/44 (2.3%)	0/44 (0.0%)	1.000	2/29 (6.9%)	0/29 (0.0%)	0.491
Vitamin D	≥Ref. *	2/33 (6.1%)	8/33 (24.2%)	0.082	13/41 (31.7%)	1/41 (2.4%)	<0.001	8/50 (16.0%)	10/50 (20.0%)	0.795	5/51 (9.8%)	9/51 (17.6%)	0.389	6/44 (13.6%)	1/44 (2.3%)	0.050	9/29 (31.0%)	4/29 (13.8%)	0.207
Calcium	≥Ref. *	1/33 (3.0%)	3/33 (9.1%)	0.613	0/41 (0.0%)	2/41 (4.9%)	0.494	0/50 (0.0%)	3/50 (6.0%)	0.242	0/51 (0.0%)	1/51 (2.0%)	1.000	2/44 (4.5%)	2/44 (4.5%)	1.000	0/29 (0.0%)	0/29 (0.0%)	1.000
Sodium	≤UL	30/33 (90.9%)	5/33 (15.2%)	<0.001	35/41 (85.4%)	10/41 (24.4%)	<0.001	36/50 (72.0%)	1/50 (2.0%)	<0.001	44/51 (86.3%)	11/51 (21.6%)	<0.001	37/44 (84.1%)	7/44 (15.9%)	<0.001	25/29 (86.2%)	6/29 (20.7%)	<0.001

Values are presented as percentages of participants meeting nutrient adequacy criteria. Adequacy was defined according to the Taiwan Dietary Reference Intakes (DRIs, 8th edition, 2022), including energy ≥ estimated energy requirement (EER), protein ≥ recommended dietary allowance (RDA), carbohydrate ≥ estimated average requirement (EAR), dietary fiber and potassium ≥ adequate intake (AI), sodium ≤ tolerable upper intake level (UL), and total fat within the acceptable macronutrient distribution range (AMDR; 25–35% of total energy intake). RCIs, residential care institutions. The complete results for all the nutrients assessed are available in Supplementary Table S3. *p*-values for nutrient adequacy comparisons were adjusted using the Benjamini–Hochberg false discovery rate (BH-FDR) procedure. Ref. *: reference intake value according to the Taiwan Dietary Reference Intakes (DRIs).

## Data Availability

The data presented in this study are available on request from the corresponding author due to ethical and privacy restrictions.

## Supplementary Materials

The following supporting information can be downloaded at https://www.mdpi.com/article/10.3390/nu18111679/s1. Table S1. Standardized mean differences before and after propensity score matching. Table S2. Conceptual mapping of selected nutrient patterns in relation to potential food sources and nutrient adequacy. Table S3. Comparison of nutrient adequacy between adolescents residing in residential care institutions (RCIs) and the NAHSIT 2010–2012 comparison group, stratified by sex and age group.
