# Supplementary material for: Investigation of Dietary Intake and Nutrient Adequacy of Adolescents in Institutional Care by Comparing with National Survey Data in Taiwan: A Cross-Sectional Study
Source: Nutrients. 2026 May 24;18(11):1679. doi: 10.3390/nu18111679 (PMC13258388; doi:10.3390/nu18111679)
Supplement: Supplementary file 1 [file nutrients-18-01679-s001.zip › nutrients-4285672-supplementary.pdf]

**Table S1. Standardized mean differences before and after propensity score matching.**

| Variable | Before Matching (SMD) | After Matching (SMD) |
|---|---|---|
| Age group | 0.10 | 0.02 |
| Sex | 0.04 | 0.01 |
| Geographic region | 0.14 | 0.03 |

Values are presented as standardized mean differences (SMDs). Covariate balance was assessed before and after propensity score matching. SMD values < 0.1 were considered indicative of an acceptable balance.

**Table S2. Conceptual mapping of selected nutrient patterns in relation to potential food sources and nutrient adequacy.**

| Nutrient Pattern (Observed) | Possible Food Source Interpretation |
|---|---|
| Lower calcium/vitamin D than NAHSIT and below the recommended levels | Suggests insufficient intake of dairy products (e.g., milk, yogurt, cheese) |
| Dietary fiber higher than NAHSIT but below recommended levels | May reflect a relatively greater intake of plant-based foods compared with NAHSIT, although overall intake remains inadequate. |
| Low potassium (below recommended levels) | Suggests insufficient intake of fruits, vegetables, and legumes |
| Low iron/zinc | May reflect limited intake of animal-source foods and other protein-rich foods (e.g., meat, seafood, legumes) |

Note: This table provides a conceptual mapping of selected nutrient patterns to potential food sources to aid interpretation of the study findings. The mapping is based on established nutritional knowledge and relevant literature, and is intended to support interpretation rather than to represent direct evidence derived from the present data. Therefore, the suggested associations should be interpreted with appropriate caution.

**Table S3. Comparison of nutrient adequacy between adolescents residing in residential care institutions (RCIs) and the NAHSIT 2010–2012 comparison group, stratified by sex and age group.**

| | | RCI | NAHSIT | *p* | RCI | NAHSIT | *p* | RCI | NAHSIT | *p* | RCI | NAHSIT | *p* | RCI | NAHSIT | *p* | RCI | NAHSIT | *p* |
|---|---|---|---|---|---|---|---|---|---|---|---|---|---|---|---|---|---|---|---|
| Energy | ≥ EER | 7/33 (21.2%) | 13/33 (39.4%) | 0.180 | 6/41 (14.6%) | 9/41 (22.0%) | 0.569 | 6/50 (12.0%) | 28/50 (56.0%) | <0.001 | 12/51 (23.5%) | 16/51 (31.4%) | 0.506 | 6/44 (13.6%) | 16/44 (36.4%) | 0.025 | 7/29 (24.1%) | 10/29 (34.5%) | 0.565 |
| Protein | ≥RDA | 14/33 (42.4%) | 27/33 (81.8%) | 0.002 | 19/41 (46.3%) | 31/41 (75.6%) | 0.012 | 17/50 (34.0%) | 42/50 (84.0%) | <0.001 | 18/51 (35.3%) | 34/51 (66.7%) | 0.003 | 9/44 (20.5%) | 32/44 (72.7%) | <0.001 | 9/29 (31.0%) | 22/29 (75.9%) | 0.001 |
| Fat | AMDR 25–35% | 10/33 (30.3%) | 13/33 (39.4%) | 0.606 | 12/41 (29.3%) | 18/41 (43.9%) | 0.252 | 19/50 (38.0%) | 21/50 (42.0%) | 0.838 | 18/51 (35.3%) | 17/51 (33.3%) | 1.000 | 15/44 (34.1%) | 15/44 (34.1%) | 1.000 | 7/29 (24.1%) | 10/29 (34.5%) | 0.565 |
| Carbohydrate | ≥ EAR | 33/33 (100.0%) | 32/33 (97.0%) | 1.000 | 41/41 (100.0%) | 41/41 (100.0%) | 1.000 | 50/50 (100.0%) | 50/50 (100.0%) | 1.000 | 51/51 (100.0%) | 49/51 (96.1%) | 0.495 | 44/44 (100.0%) | 44/44 (100.0%) | 1.000 | 26/29 (89.7%) | 29/29 (100.0%) | 0.237 |
| Dietary fiber | ≥ AI | 2/33 (6.1%) | 1/33 (3.0%) | 1.000 | 1/41 (2.4%) | 1/41 (2.4%) | 1.000 | 1/50 (2.0%) | 0/50 (0.0%) | 1.000 | 3/51 (5.9%) | 2/51 (3.9%) | 1.000 | 1/44 (2.3%) | 0/44 (0.0%) | 1.000 | 2/29 (6.9%) | 0/29 (0.0%) | 0.491 |
| Vitamin A | ≥ Ref.* | 17/33 (51.5%) | 21/33 (63.6%) | 0.455 | 24/41 (58.5%) | 23/41 (56.1%) | 1.000 | 25/50 (50.0%) | 28/50 (56.0%) | 0.689 | 30/51 (58.8%) | 24/51 (47.1%) | 0.321 | 21/44 (47.7%) | 15/44 (34.1%) | 0.278 | 18/29 (62.1%) | 9/29 (31.0%) | 0.034 |
| Vitamin D | ≥ Ref.* | 2/33 (6.1%) | 8/33 (24.2%) | 0.082 | 13/41 (31.7%) | 1/41 (2.4%) | <0.001 | 8/50 (16.0%) | 10/50 (20.0%) | 0.795 | 5/51 (9.8%) | 9/51 (17.6%) | 0.389 | 6/44 (13.6%) | 1/44 (2.3%) | 0.050 | 9/29 (31.0%) | 4/29 (13.8%) | 0.207 |
| Vitamin E | ≥ Ref.* | 21/33 (63.6%) | 10/33 (30.3%) | 0.013 | 18/41 (43.9%) | 9/41 (22.0%) | 0.059 | 18/50 (36.0%) | 16/50 (32.0%) | 0.833 | 17/51 (33.3%) | 7/51 (13.7%) | 0.034 | 17/44 (38.6%) | 8/44 (18.2%) | 0.057 | 9/29 (31.0%) | 4/29 (13.8%) | 0.207 |
| Vitamin C | ≥ Ref.* | 22/33 (66.7%) | 16/33 (48.5%) | 0.213 | 16/41 (39.0%) | 20/41 (48.8%) | 0.505 | 21/50 (42.0%) | 24/50 (48.0%) | 0.688 | 25/51 (49.0%) | 20/51 (39.2%) | 0.425 | 18/44 (40.9%) | 16/44 (36.4%) | 0.827 | 11/29 (37.9%) | 13/29 (44.8%) | 0.790 |
| Vitamin B1 | ≥ Ref.* | 1/33 (3.0%) | 20/33 (60.6%) | < 0.001 | 0/41 (0.0%) | 15/41 (36.6%) | <0.001 | 0/50 (0.0%) | 33/50 (66.0%) | <0.001 | 3/51 (5.9%) | 24/51 (47.1%) | < 0.001 | 2/44 (4.5%) | 28/44 (63.6%) | <0.001 | 3/29 (10.3%) | 13/29 (44.8%) | 0.007 |
| Vitamin B2 | ≥ Ref.* | 2/33 (6.1%) | 21/33 (63.6%) | < 0.001 | 2/41 (4.9%) | 17/41 (41.5%) | <0.001 | 6/50 (12.0%) | 29/50 (58.0%) | <0.001 | 10/51 (19.6%) | 27/51 (52.9%) | < 0.001 | 7/44 (15.9%) | 15/44 (34.1%) | 0.084 | 3/29 (10.3%) | 13/29 (44.8%) | 0.007 |
| Niacin | ≥ Ref.* | 1/33 (3.0%) | 25/33 (75.8%) | < 0.001 | 1/41 (2.4%) | 23/41 (56.1%) | <0.001 | 4/50 (8.0%) | 36/50 (72.0%) | <0.001 | 3/51 (5.9%) | 26/51 (51.0%) | < 0.001 | 2/44 (4.5%) | 28/44 (63.6%) | <0.001 | 1/29 (3.4%) | 12/29 (41.4%) | <0.001 |
| Vitamin B6 | ≥ Ref.* | 9/33 (27.3%) | 23/33 (69.7%) | 0.001 | 7/41 (17.1%) | 22/41 (53.7%) | <0.001 | 16/50 (32.0%) | 40/50 (80.0%) | <0.001 | 11/51 (21.6%) | 27/51 (52.9%) | 0.002 | 7/44 (15.9%) | 26/44 (59.1%) | <0.001 | 5/29 (17.2%) | 15/29 (51.7%) | 0.012 |
| Vitamin B12 | ≥ Ref.* | 16/33 (48.5%) | 27/33 (81.8%) | 0.009 | 7/41 (17.1%) | 25/41 (61.0%) | <0.001 | 11/50 (22.0%) | 38/50 (76.0%) | <0.001 | 12/51 (23.5%) | 33/51 (64.7%) | <0.001 | 4/44 (9.1%) | 22/44 (50.0%) | <0.001 | 4/29 (13.8%) | 19/29 (65.5%) | <0.001 |
| Calcium | ≥ Ref.* | 1/33 (3.0%) | 3/33 (9.1%) | 0.613 | 0/41 (0.0%) | 2/41 (4.9%) | 0.494 | 0/50 (0.0%) | 3/50 (6.0%) | 0.242 | 0/51 (0.0%) | 1/51 (2.0%) | 1.000 | 2/44 (4.5%) | 2/44 (4.5%) | 1.000 | 0/29 (0.0%) | 0/29 (0.0%) | 1.000 |
| Iron | ≥ Ref.* | 6/33 (18.2%) | 24/33 (72.7%) | < 0.001 | 7/41 (17.1%) | 16/41 (39.0%) | 0.048 | 14/50 (28.0%) | 36/50 (72.0%) | <0.001 | 11/51 (21.6%) | 22/51 (43.1%) | 0.033 | 15/44 (34.1%) | 26/44 (59.1%) | 0.032 | 5/29 (17.2%) | 8/29 (27.6%) | 0.530 |
| Potassium | ≥ AI | 2/33 (6.1%) | 16/33 (48.5%) | < 0.001 | 5/41 (12.2%) | 14/41 (34.1%) | 0.035 | 8/50 (16.0%) | 26/50 (52.0%) | <0.001 | 4/51 (7.8%) | 17/51 (33.3%) | 0.003 | 6/44 (13.6%) | 13/44 (29.5%) | 0.126 | 6/29 (20.7%) | 5/29 (17.2%) | 1.000 |
| Magnesium | ≥ Ref.* | 8/33 (24.2%) | 21/33 (63.6%) | 0.003 | 8/41 (19.5%) | 13/41 (31.7%) | 0.312 | 4/50 (8.0%) | 19/50 (38.0%) | <0.001 | 6/51 (11.8%) | 10/51 (19.6%) | 0.415 | 2/44 (4.5%) | 10/44 (22.7%) | 0.026 | 2/29 (6.9%) | 4/29 (13.8%) | 0.670 |
| Phosphorus | ≥ Ref.* | 8/33 (24.2%) | 26/33 (78.8%) | < 0.001 | 5/41 (12.2%) | 26/41 (63.4%) | <0.001 | 9/50 (18.0%) | 43/50 (86.0%) | <0.001 | 7/51 (13.7%) | 27/51 (52.9%) | < 0.001 | 7/44 (15.9%) | 32/44 (72.7%) | <0.001 | 4/29 (13.8%) | 13/29 (44.8%) | 0.020 |
| Zinc | ≥ Ref.* | 2/33 (6.1%) | 23/33 (69.7%) | < 0.001 | 1/41 (2.4%) | 20/41 (48.8%) | <0.001 | 0/50 (0.0%) | 26/50 (52.0%) | <0.001 | 3/51 (5.9%) | 16/51 (31.4%) | 0.002 | 0/44 (0.0%) | 13/44 (29.5%) | <0.001 | 3/29 (10.3%) | 7/29 (24.1%) | 0.297 |
| Sodium | ≤ UL | 30/33 (90.9%) | 5/33 (15.2%) | < 0.001 | 35/41 (85.4%) | 10/41 (24.4%) | <0.001 | 36/50 (72.0%) | 1/50 (2.0%) | <0.001 | 44/51 (86.3%) | 11/51 (21.6%) | < 0.001 | 37/44 (84.1%) | 7/44 (15.9%) | <0.001 | 25/29 (86.2%) | 6/29 (20.7%) | <0.001 |

Data are presented as n/N (%). Nutrient adequacy was defined according to the Dietary Reference Intakes (DRIs) for Taiwanese populations. Energy adequacy was defined as intake ≥ estimated energy requirement (EER); protein adequacy as intake ≥ recommended dietary allowance

(RDA); carbohydrate adequacy as intake ≥ estimated average requirement (EAR); dietary fiber and potassium adequacy as intake ≥ adequate intake (AI); sodium adequacy as intake ≤ tolerable upper intake level (UL); and fat adequacy as intake within the acceptable macronutrient distribution range (AMDR, 25–35% of total energy intake). Vitamin A intake was expressed as retinol equivalents (RE) according to the food composition database used for nutrient ana
